# Nutrition, Nutritional Status, Micronutrients Deficiency, and Disease Course of Inflammatory Bowel Disease

**DOI:** 10.3390/nu15173824

**Published:** 2023-08-31

**Authors:** Marco Valvano, Annalisa Capannolo, Nicola Cesaro, Gianpiero Stefanelli, Stefano Fabiani, Sara Frassino, Sabrina Monaco, Marco Magistroni, Angelo Viscido, Giovanni Latella

**Affiliations:** 1 Gastroenterology Unit, Division of Gastroenterology, Hepatology, and Nutrition, Department of Life, Health and Environmental Sciences, University of L’Aquila, Piazzale Salvatore Tommasi 1, 67100 L’Aquila, Italy; dott.nicolacesaro@gmail.com (N.C.); sfabiani92@gmail.com (S.F.); sara.frassino@gmail.com (S.F.); monaco.sa6@gmail.com (S.M.); magistroni.marco@gmail.com (M.M.); angelo.viscido@univaq.it (A.V.); giovanni.latella@univaq.it (G.L.); 2 Division of Gastroenterology, Galliera Hospital, 16128 Genoa, Italy; giastefanelli@gmail.com; 3 Diagnostic and Surgical Endoscopy Unit, San Salvatore Academic Hospital, 67100 L’Aquila, Italy; annalisacap@tiscali.it

**Keywords:** inflammatory bowel disease, nutrition, micronutrients, anemia, sarcopenia, obesity

## Abstract

During the disease course, most Inflammatory Bowel Disease patients present a condition of malnutrition, undernutrition, or even overnutrition. These conditions are mainly due to suboptimal nutritional intake, alterations in nutrient requirements and metabolism, malabsorption, and excessive gastrointestinal losses. A suboptimal nutritional status and low micronutrient serum levels can have a negative impact on both induction and maintenance of remission and on the quality of life of Inflammatory Bowel Disease patients. We performed a systematic review including all the studies evaluating the connection between nutrition, nutrition status (including undernutrition and overnutrition), micronutrient deficiency, and both disease course and therapeutic response in Inflammatory Bowel Disease patients. This systematic review was performed using PubMed/MEDLINE and Scopus. Four main clinical settings concerning the effect of nutrition on disease course in adult Inflammatory Bowel Disease patients were analyzed (induction of remission, maintenance of remission, risk of surgery, post-operative recurrence, and surgery-related complications). Four authors independently reviewed abstracts and manuscripts for eligibility. 6077 articles were found; 762 duplicated studies were removed. Out of 412 full texts analyzed, 227 were included in the review. The evidence summarized in this review showed that many nutritional aspects could be potential targets to induce a better control of symptoms, a deeper remission, and overall improve the quality of life of Inflammatory Bowel Disease patients.

## 1. Introduction

Inflammatory bowel diseases (IBD) are chronic inflammatory diseases, mediated by the immune system, which affect the gastrointestinal tract. The two main manifestations are Crohn’s Disease (CD) and ulcerative colitis (UC). CD may affect any area of the gastrointestinal tract and has a transmural involvement. UC generally occurs only in the rectum and colon and involves the mucosa and submucosa layers [1,2].

The etiology of IBD is not completely defined. Yet, several studies support the hypothesis that their onset is due to a combination and interplay of genetic factors, immune dysregulation and environmental triggers that can modify gut microbiome [3,4,5]. In this scenario, diet is a potential environmental trigger. The global increasing incidence of IBD seems to be associated with Western lifestyle and diet: a high intake of proteins and red meat can result in an increased production of bacterial metabolites, such as an increase of ammonia, indoles, phenols, and sulphides, and a decrease of short-chain fatty acids (SCFAs), which could all be involved in the development of IBD [6,7].

As a consequence, diet modifications have been considered therapeutic tools; for example, in pediatric IBD patients, enteral nutrition has been shown to be effective in inducing clinical remission, independently of the used formula [8,9,10].

Malnutrition, undernutrition and overnutrition seen in such patients are variable during the disease course [11] and due to suboptimal nutritional intake, alterations in nutrient requirements and metabolism, malabsorption, excessive gastrointestinal losses, and medication [12].

At the time of diagnosis, 60% of CD patients and 35% of UC patients are underweight, even if this proportion is lowered in the last years, reflecting the increased incidence of obesity [13,14]: 20–40% of adult patients with IBD are overweight (25 < body mass index (BMI) < 30 kg/m^2^), and an additional 15–40% are obese (BMI > 30 kg/m^2^) [15].

Obesity is associated with treatment failure (especially with anti-TNF drugs), risk of hospitalization, and lower endoscopic remission rates [16,17,18,19]; sarcopenia in overweight IBD patients (BMI ≥ 25 kg/m^2^) is the only significant predictor of the need for surgery (*p* = 0.002) [20]; nutritional deficits and low micronutrients serum levels can have a negative impact on both induction and maintenance of remission and on the quality of life of these patients [21,22]. Thus, the assessment of nutritional status in IBD patients is a crucial issue and current guidelines suggest that patients with IBD should be regularly screened for nutritional status, micronutrient deficiencies and bone mineral density [21,23].

Currently, there are limited data on the disease course and therapy response in cases of malnutrition in IBD, especially in the context of sarcopenia and undernutrition. Moreover, if micronutrient or vitamin supplementation (e.g., vitamin D supplementation) could be a potential therapeutic option or only an effect of disease activity it is still unclear [24,25].

The aim of this systematic review is trying to clarify the connection between nutrition, malnutrition (including overnutrition and undernutrition), micronutrient deficiency, and both disease course and therapeutic response in IBD patients.

## 2. Materials and Methods

This systematic review was performed using PubMed/MEDLINE and Scopus. For each of the relevant publications (previous review articles and included studies), reference sections were also screened for other applicable publications.

The research strategy for each clinical question is reported in the Supplementary Material (Table S1). We found 6077 articles; 762 duplicated studies were removed. Out of 412 full texts analyzed, 227 were included in the review (Figure S1).

No filters were used in the search strategy. The data of the last search was May 2023. The complete selection process is reported in the Supplementary Material (Table S2).

Four authors did a systematic literature search. Clinical questions and related outcomes of interest were identified using the PICO framework. Four main clinical settings concerning adult IBD patients were identified.

- Induction of remission
- Maintenance of remission
- Risk of surgery
- Postoperative recurrence (POR) and surgery-related complications

### 2.1. Selection of Studies and Data Extraction

Four authors (GS, SF, SM, and MM) independently reviewed abstracts and manuscripts for eligibility.

Conflicts were resolved by consensus, referring to the original articles. The selection was made according to the following criteria:

### 2.2. Inclusion CriteriaPatient Type: Adult Patients (age ≥ 18) with a Confirmed Diagnosis of IBD

- Intervention: Nutritional management; Nutritional evaluation; serum evaluation or supplementation of micronutrients or albumin.
- Outcome: evaluation of clinical relapse or disease activity (evaluated with disease activity score or loss of response to therapy); risk of surgery; POR and surgery-related complications
- Study type: Meta-analysis, Randomized clinical trial (RCT), Non-randomized study of intervention (NRSI), cross-sectional study.

### 2.3. Exclusion Criteria

- Paediatric patients
- Non-human study
- Lack of data concerning clinical response, risk of surgery, POR, and surgery-related complications.

Four reviewers (GS, SF, SM, and MM) independently reviewed the literature according to the above-predefined strategy and criteria and selected eligible studies; any disagreement was resolved by consensus or by recourse to a fifth author (MV).

Each reviewer extracted the data of interest in a pre-made template: title and reference details (first author, journal, year, country), study population characteristics (number of patients, gender, age, disease type (UC or CD), intervention details and outcome data (induction of remission, maintenance of remission, risk of surgery, POR, and surgery-related complications).

All data were recorded independently by the literature reviewers in separate databases and will be compared at the end of the reviewing process to limit selection bias. The database was also reviewed by another author (MV). Any disagreement was resolved by consensus or by recourse to the senior author.

## 3. Nutrition and Nutritional Status

If food is implicated in the pathogenesis of IBD is still not clear, but the impact of some nutrients on the behalf of the gastrointestinal tract has been suggested. For example, dietary fibre escape digestion in the small bowel and enter the colon where they are metabolized by gut bacteria which produce SCFAs, energy sources for colonocytes [26]. On the contrary red meat and other high-protein foods, contribute to sulphides formation which damages the mucus in the colon [27,28]. In the IBD population, 70% of patients report food-related symptom exacerbation, while a wide variety of foods are believed to be helpful [29]. This becomes a concern when patients drastically reduce or completely avoid important nutrients such as folic acid, calcium, vitamin B 12, and iron which represent the most frequent nutritional deficiency in IBD patients. This attitude, summed to disease duration, extent and severity, may put them at risk of developing nutritional deficiencies in the long term [30].

### 3.1. Nutrition and Exclusion Diet

Compared to recommended requirements, adults with IBD have an inadequate intake of energy, fibres, fat-soluble vitamins, folate and calcium [31]. Nutritional support for correcting deficiencies can be provided summarily in the form of parenteral nutrition (PN), enteral nutrition (EN) and specific diets.

#### 3.1.1. Induction of Remission

Literature evidence seems to agree that steroids are more effective than EN in inducing remission in CD; anyway, both PN and EN used in combination with steroids, can improve the response rate to intravenous corticosteroid therapy [32,33,34,35,36,37]. Regarding UC, there is no evidence that EN alters the inflammatory response: therefore, it is not used in the treatment of active disease nor in the maintenance of remission in UC [38] (Table 1).

Oral diets proposed for active UC have been analyzed in the recent meta-analysis from Limketkai: symptom-guided exclusion diet, milk protein elimination, and gluten-free diet were considered. No one seems to be helpful for the induction of remission [48]. Recently, a UC Exclusion Diet (UCED), already proposed for a paediatric population and composed of limited animal fats and sulphated amino acids consumption, has been applied in a blinded, randomized controlled (CRAFT UC) including adults patients with active UC refractory to therapy; patients receiving UCED alone achieved higher remission and mucosal healing rates compared to those undergoing to faecal transplantation, with or without dietary modification [45,46].

Similarly, an open-label, pilot randomised trial shows that the CD Exclusion Diet (CDED), with or without partial EN, is effective for induction and maintenance of remission in biologic naive adults with mild-to-moderate CD [47]. Also, low refined carbohydrate diet and symptoms-guided diet outperformed controls for induction of remission in CD, even if with very low certainty of evidence [48].

Regarding the habit of reducing fibres in the acute phase, a prospective randomized controlled study on active CD concluded that consuming a low residue diet gave no advantage in terms of symptoms, hospitalization, surgery and nutritional status compared to a free Italian-style diet—rich in fibres [41]. Similar conclusions were more recently achieved in the DINE-CD trial on 191 CD patients with mild to moderate symptoms: leaving a Mediterranean diet in favor of the so-called Specific Carbohydrate Diet (SCD) eliminating all grains, sugars, milk products and most processed foods, gives no advantage in terms of clinical and biochemical remission [49].

#### 3.1.2. Maintenance of Remission

Two meta-analyses focused on the use of EN in CD patients in clinical remission, both regarding partial EN, offering the possibility to add an oral daily diet. The type of enteral formulation (elemental, semi-elemental or polymeric) does not make a difference in terms of efficacy [33,34] (Table 1). The first, dated 2015, assessed that the combination of infliximab therapy plus a specialized EN is more effective in achieving and maintaining clinical remission compared with infliximab monotherapy. However, only 4 studies were included, all Japanese, the majority were retrospective and evaluating only the subjective clinical outcome [39]. More convincing evidence comes from another meta-analysis on 429 CD patients in remission and on maintaining therapy; 224 received EN and 205 received non-EN treatment as a control treatment. Patients receiving EN exhibited a higher frequency of clinical remission maintenance at 0.5–1 year and a significantly lower rate of clinical relapse at 0.5–2 years [40].

Fibre consumption seems to be beneficial also during remission. Two Japanese prospective, single-centre trials conclude that a semi-vegetarian diet, added to maintenance therapy, has a protective effect against relapse both in CD and UC [42,43]. In an open-label, parallel-group, multicentre, randomized clinical trial, UC patients in remission receiving only fermentable fibres, has the same relapse rate as those receiving mesalamine or mesalamine plus fibres [44].

It is not clear if reducing meat could be useful. While a prospective cohort study on 191 UC patients shows that a higher intake of meat may increase the risk of a relapse, the reduction of red meat as well as refined carbohydrates did not reduce the risk of relapse for CD [27,48]. The carrageenan-free diet, the anti-inflammatory diet and the milk protein elimination diet have no impact on the maintenance of remission in UC [48].

#### 3.1.3. Risk of Surgery

A poor preoperative nutritional status is associated with an increased risk of postoperative complications [50]. If oral feeding is not sufficient, guidelines recommend that EN should be preferred over PN in malnourished patients and to postpone if possible surgery for 7–14 days to correct malnutrition; PN should be used as a supplement to EN if >60% of energy needs cannot be met via the enteral route or when an enteral administration is not possible (obstruction, anastomotic leak, fistula) [51].

Data arising from a meta-analysis suggest that CD patients receiving preoperative EN or PN are 74% less likely to have a postoperative complication compared to those receiving standard care without nutritional support; in particular, EN is significantly superior to standard of care without nutrition support in reducing post-operative complications, while PN has a trend toward being superior to standard [52]. However, other authors conducted systematic reviews on this topic and although all studies included presented encouraging results on nutritional support, both did not conduct a meta-analysis because of the heterogeneity of the studies, most classified as medium-low quality [53,54] (Table 2).

#### 3.1.4. Post-Operative Recurrence and Surgery-Related Complications

Two post-surgical scenarios in which nutrition can be pivotal are pouchitis and short bowel syndrome, but quality studies are lacking. Regarding pouchitis, the literature reports several dietary interventions in small numbers of patients, and some of these studies were retrospective and without controls.

We only found a prospective study testing exclusive EN given for 28 days to 7 patients with chronic antibiotic-dependent pouchitis, resulting in a symptomatic improvement but no reduction in endoscopic or histological signs of inflammation [55].

Consumption of fibres has been considered in patients with a pouch, either as adjunctive fibre supplementation (e.g., inulin), or as a fibre-rich diet. A randomized, double-blind, placebo-control crossover study on 20 patients with J-pouch, revealed that dietary inulin supplementation leads to a reduction of endoscopic and histologic inflammation of pouch mucosa [56]. A prospective cross-sectional study compared the dietary intake of 80 pouch patients to that of 80 healthy volunteer controls; within the study population, it compared those with a normal pouch to those with pouchitis. Pouch patients consumed significantly higher servings of fats and oils compared to healthy controls; patients with pouchitis consume fewer fruit servings and antioxidants than patients with a normal pouch [57]. Godny and colleagues suggest that consuming ≥1.5 fruit servings/day would be associated with a reduced risk of developing pouchitis in the following year [58].

Short bowel syndrome is a malabsorption syndrome consequence of multiple intestinal resections, whose severity and clinical presentation depending on the site and the extension of the resection, the health of the remaining mucosa and its ability to compensate. Intestinal failure resulting, can be severe, moderate or mild, requiring respectively PN, EN or oral supplements/dietary adjustments [60]. However, literature evidence in this field is limited, due to the extreme variety of presentations and the scarcity of IBD-specific works. ESPEN guidelines on chronic intestinal failure in adults recommend that patients with short bowel syndrome with a preserved colon consume a high-carbohydrate-low-fat diet; instead, the fat: carbohydrate ratio seems of less importance in patients without a colon, although the grade of evidence is low [59], being literature about this topic discordant [61,62]. A meta-analysis of clinical trials showed that the treatment with growth hormone and glutamine combined with a modified high-carbohydrate-low-fat diet was effective without any major adverse effects in patients with short bowel syndrome [63].

#### 3.1.5. Main Evidence and Clinical Implications

- PN and EN used in combination with steroids, can improve the response rate to intravenous corticosteroid therapy.
- CDED is effective for induction and maintenance of remission in mild-to-moderate, biologic naive CD.
- Only a low refined carbohydrate diet and a symptoms-guided diet outperformed controls for induction of remission in CD, even if with very low certainty of evidence.
- In CD patients in maintenance therapy, adding EN offers better results in maintaining clinical remission. In CD and UC patients in maintenance therapy high fibre diets have a protective effect against relapse.
- Preoperative EN or PN reduce the risk of postoperative complication in CD patients.
- Exclusive EN in patients with chronic antibiotic dependent pouchitis can improve symptoms but not endoscopic or histological signs of inflammation; fibre intake can reduce endoscopic and histologic inflammation of pouch mucosa and reduce the risk of pouchitis.

### 3.2. Sarcopenia

Sarcopenia is defined by a loss in muscle mass and lean body mass that leads to functional changes and decreased strength [64].

The European Working Group on Sarcopenia in Older People (EWGSOP) developed 3 criteria for its definition and diagnosis: low muscle mass, low muscle strength, low physical performance (being necessary for the diagnosis the first criterion, plus one or both of the other two criteria) [65].

During disease flares, both the reduced caloric intake and the mucosa inflammation can impair nutrient absorption and determine weight loss [21].

#### 3.2.1. Clinical Disease Course and Response to Therapies

Most of the studies comparing the course of CD in sarcopenic patients versus non-sarcopenic, showed a worse disease course in the first group, in terms of the need for corticosteroids, the occurrence of complications, fecal calprotectin (FC) value, and endoscopic recurrence [66,67,68,69,70,71,72,73]; in most of the studies such difference was statistically significant, while for just two of them a trend to a worse course emerged [74,75].

#### 3.2.2. Risk of Surgery

In a meta-analysis published in 2017 including eight non-randomized studies, the authors showed that 46.2% (409/885) of IBD patients presented a condition of sarcopenia, with a higher prevalence in CD compared to UC (571 and 341 patients, respectively) [76]. This meta-analysis reported a non-significant difference between the sarcopenic and non-sarcopenic groups in terms of the need for surgery (unadjusted OR: 1.826; 95% CI 0.913–3.654; *p* = 0.089; I2 = 54.62%, *p* = 0.051) and post-operative complications (unadjusted OR: 3.265; 95% CI 0.575–18.557; *p* = 0.182; I2 = 88.46%, *p* < 0.001); however, pooled adjusted data for significant covariates among three included studies showed that sarcopenic group presented higher prevalence of surgery (adjusted OR: 2.665; 95% CI 1.121–6.336; *p* = 0.027; I2 = 33.94%, *p* = 0.220) and post-operative complications (adjusted OR = 6.097; 95% CI 1.756–21.175; *p* = 0.004; I2 = 0.0%, *p* = 0.637) [76]. Such a higher incidence of surgery in sarcopenic IBD patients is also reported by two observational studies, where higher odds of requiring surgery within 1 year were assessed also in those on biological therapy (OR, 2.36; 95% CI, 1.06–5.26, *p* = 0.04) [77,78].

Regarding CD, some authors [66,75,79,80,81] reported a higher incidence of surgery and shorter surgery-free survival in sarcopenic patients compared to non-sarcopenic, while others do not [73,82,83,84]. Bamba et al. concluded that sarcopenia is a risk factor for surgery in active CD patients, in univariate and multivariate analysis (OR 0.313; *p* = 0.006, OR 0.318; *p* = 0.015) [80].

Concerning UC patients, we found three retrospective studies including hospitalized patients with acute severe ulcerative colitis (ASUC). The need for rescue therapy and colectomy was higher for sarcopenic than non-sarcopenic patients (*p* < 0.001 and *p* = 0.02 for rescue therapy; *p* = 0.001 and *p* = 0.16 for colectomy) [85,86]. Moreover, sarcopenia results an independent risk factor for rescue therapy (OR 4.079, *p* < 0.001; OR 3.401, *p* = 0.03), urgent surgery (*p* = 0.001 and OR 2.999, 95% CI 1.285–6.997; *p* = 0.01) and post-operative complications (*p* = 0.003; OR 4.157, 95% CI 1.364–12.667, *p* = 0.012, respectively) in univariate and multivariate analysis [85,87].

#### 3.2.3. Post-Operative Recurrence and Surgery-Related Complications

Other recent studies not included in the above mentioned meta-analysis reported a higher incidence of post-operative complications and a higher risk of post-surgery re-hospitalization in sarcopenic IBD patients [78,88,89,90], except one [91].

Considering CD, five studies showed a worse post-operative course (evaluated through various outcomes) in sarcopenic patients [72,84,92,93,94], while two studies did not confirm such differences [74,95].

Concerning UC, sarcopenia seems to be an independent risk factor for surgical site infections after ileal pouch-anal anastomosis (IPAA) in univariate and multivariate analysis (OR 5.85; *p* = 0.008 and OR 4.91; *p* = 0.03, respectively) [96].

In conclusion, sarcopenia seems to be a relevant predictor of a worse disease course. It is important to evaluate the presence of sarcopenia, especially in patients with severe disease, the elderly, and patients with risk-factors for surgery. In these groups, sarcopenia can increase the need for rescue therapy, the need surgery or urgent surgery, and even post-surgery complications.

#### 3.2.4. Main Evidence and Clinical Implications

- Sarcopenia in CD patients may result in a slight increase of worse clinical, biochemical, and endoscopic outcomes.
- In IBD patients, in CD patients and ASUC setting sarcopenia results in a slight increase in the need for surgery, shorter surgery-free time or colectomy.
- In IBD patients, in CD patients and after the IPAA setting, sarcopenia is a risk factor for post-operative complications.

### 3.3. Obesity

In the last decades, overweight (BMI > 25 kg/m^2^) and obesity (BMI > 30 kg/m^2^) increased not only in the general population but also among IBD patients [97]. In a time-trend analysis involving 10,282 CD patients (from 1991 to 2008) and including 40 RCT, the mean BMI increased from 20.8 to 27.0 [98]. Obesity and overweight were reported respectively in 18% and 38% of IBD patients, with a higher percentage of obesity in CD than in UC [99].

Only few studies analyzed the impact of obesity on the course of UC, while more data are available in CD patients. Anyhow, results are often contrasting and non-conclusive [100]. Obesity may be associated with a worse prognosis in CD in terms of perianal complications, disease activity, hospitalization, time to the first surgery and more aggressive medical treatment [101]. This could be probably due to the ability of visceral fat to produce cytokines and thus promote inflammation [102].

#### 3.3.1. Clinical Disease Course and Response to Therapies

Obesity and overweight seem to negatively impact the efficacy of medications [103].

Obese patients treated with thiopurines and anti-TNF could have worse outcomes compared to non-obese patients, due to subtherapeutic dosing: a retrospective study among 1494 IBD patients (634 UC, 860 CD) found a lower dosing per kilogram of AZA, 6-MP, methotrexate and anti-TNF in the obese subgroup (*p* < 0.0001) [104].

A retrospective analysis conducted on CD patients revealed that during the first year of therapy with IFX, clinical flare, loss of response and need for surgery are improved by BMI increase, but extreme values (BMI < 18.5 kg/m^2^ and BMI > 30 kg/m^2^) are associated with worse outcomes [17]. Moreover, obese patients starting IFX may also have a higher risk of flare (HR 3.03, *p* < 0.001), with an earlier flare the greater the BMI, both in CD (HR = 1.06 per 1 kg/m^2^ increase, *p* = 0.02) and in UC (HR = 1.30 per 1 kg/m^2^ increase, *p* = 0.01) [19]. Nevertheless, the literature data are not univocal.

From an analysis of pooled data among 1014 CD patients exploring prognostic factors to IFX therapy persistence, emerges that the difference in the prevalence of obesity between American and European patients did not influence long-term (>5 years) use of IFX [105]. For some authors, obesity could be a risk factor for a shorter time to dose escalation in ADA patients (*p* = 0.013), but not for IFX (*p* = 0.164) [16]. Another pooled analysis among IBD patients treated with IFX (723 CD, 484 UC) explored the influence of BMI on clinical remission, clinical response, and mucosal healing; it concluded that obesity is not associated with an inferior response to IFX (OR 0.94, *p* = 0.97; OR 0.84, *p* = 0.45; OR 1.13, *p* = 0.95, respectively) [106].

#### 3.3.2. Risk of Surgery

A review including 22 articles showed an association between visceral adiposity and both the risk of complex CD (OR 26.1; 95% CI 2–75.4, *p* = 0.02), and POR (RR 2.1; 95% CI 1.5–3; *p* = 0.012). However, data about visceral adiposity on postsurgical complications and the efficacy of medical therapy are conflicting [107].

A meta-analysis including 7 studies (5 retrospective and 2 prospective–5 with IBD patients and 2 with CD patients only) comparing obese and non-obese patients, showed no differences in the risk of perianal disease (RR = 0.97, *p* = 0.81) and use of anti-TNF (RR= 0.89, *p* = 0.26) or immunomodulators (RR = 0.96, *p* = 0.43); moreover, obese patients have a lower risk of hospitalization (RR 0.84, *p* = 0.003) and surgery (RR 0.82, *p* = 0.003). However, the authors hypothesized that obesity could be an indirect sign of remission [108].

Similar results were achieved in a cross-sectional study among 846 CD patients, concluding that there was no association between obesity and perianal disease, stricturing disease, or surgery (OR = 0.71; 95% CI 0.39–1.28), while obesity was associated with a lower risk of penetrating disease (OR = 0.56; 95% CI 0.31–0.99) [109].

A retrospective study on 148 CD patients, comparing patients with BMI < 18.5 kg/m^2^ with those with a BMI > 25 kg/m^2^, showed that the latters have a significantly shorter time to first surgery (252 versus 24 months, respectively; *p* = 0.043) [110]. Other two retrospective studies evaluating visceral adipose tissue by CT scan in CD patients, concluded that high visceral adipose subjects have an increased risk of surgery (OR = 2.02; *p* = 0.006) [111] and of complicated disease (stricturing/penetrating disease and previous surgeries) (OR 26.1; 95% CI 2.0–754; *p* = 0.02) [67].

#### 3.3.3. Post-Operative Recurrence and Surgery-Related Complications

Concerning the prognostic impact of obesity on IBD patients undergoing surgery, more data are available and quite concordant. There are two recent meta-analyses including a total of 16,933 IBD patients confirming that obese patients are at higher risk of post-surgical complication (OR = 1.45; CI 95% 1.24–1.69) [112] (OR = 1.33; CI 95% 1.04–1.70) [113]. These results seem to be confirmed even in another recent meta-analysis including a more heterogeneous population—not only IBD—undergoing colectomy and IPAA (OR 2.27; CI 95% 1.42–3.61) [114] and in three more studies not included in the above-mentioned meta-analyses [115,116,117].

A post-hoc analysis of POCER study including 44 CD patients concluded that visceral adiposity is a risk factor for endoscopic post-operative recurrence [70]. A population-based study among 143 CD patients who underwent elective ileocolectomy and studied by preoperative CT scan, found that the visceral/subcutaneous fat ratio is a more reliable predictor of post-operative morbidity than conventional adiposity markers such as BMI (*p* = 0.03) [101].

#### 3.3.4. Main Evidence and Clinical Implications

- Obesity may worsen the effectiveness of IBD therapies, but the evidence is very uncertain.
- Data concerning obesity and the risk of surgery are contrasting and the quality of evidence is low.
- The only meta-analysis concludes that obesity does not increase the risk of perianal disease, stricture disease, surgery, or the use of immunomodulators/anti-TNF therapies.
- Obesity increases post-operative complications in IBD patients and in CD is a risk factor for endoscopic post-operative recurrence.

### 3.4. Albuminemia

Chronic inflammation of the mucosa determines both malabsorption and intestinal protein losses, resulting in hypoalbuminemia.

#### 3.4.1. Clinical Disease Course and Response to Therapies

In IBD, hypoalbuminemia correlates with more relapses, with secondary loss of response to infliximab [118,119] and with increased clearance of infliximab and golimumab [120,121].

Low albumin levels are associated with longer hospitalizations and with higher rates of surgery (OR 2.54; 95% CI 1.15–3.93) [122].

In CD patients, the higher the albumin levels, the higher the clinical remission rate after a dose escalation of infliximab [123]; while hypoalbuminemia is associated with a worse outcome, a greater risk of relapse and interrupting anti-TNF therapy for albumin serum levels < 3.5 g/dL (*p* = 0.0274) [124,125,126].

Albumin levels are directly proportional to adalimumab levels and inversely proportional to anti-adalimumab antibodies [127]. Moreover, values of albuminemia < 3 g/dL correlate with worse outcomes in patients with enterocutaneous fistulas and with a higher probability of abdominal abscesses during infliximab therapy [128,129].

Hypoalbuminemia is a negative prognostic factor also for UC, associated with a more severe course [130], a greater use of steroids, a worse endoscopic response/remission and primary failure to infliximab [131,132].

#### 3.4.2. Risk of Surgery

In UC patients with hypoalbuminemia, a higher likelihood of colectomy has been shown [131,133,134]; albumin values > 3.5 g/dL are an independent factor of colectomy-free survival with OR 3.03 (95% CI, 1.12–8.22; *p* = 0.029) [135], while values < 3 g/dL (HR, 2.67; 95% CI, 1.20–5.92) are associated with an increased risk of colectomy, and values < 2.45 g/dL on admission represent a significant independent predictor of colectomy (OR 6.097, 95% CI 1.8310–20.3047) [136,137]. Increasing CPR/albumin ratio and platelet/albumin ratio are suggested by some authors as predictors of rescue therapy failure and, as a consequence, of surgery [138,139,140,141,142,143,144].

#### 3.4.3. Post-Operative Recurrence and Surgery-Related Complications

Literature evidence demonstrates a higher incidence of post-surgical complications, mortality, anastomotic leaks (OR 2.8; 95% CI 1.3–5.1; *p* = 0.03), post-operative ileus, and a higher prevalence of incisional hernia (HR 2.02, *p* = 0.002) in IBD patients with hypoalbuminemia (OR 2.72; *p* = 0.03) [145,146,147,148,149].

In particular, for UC patients a greater risk of portal-mesenteric thrombosis and infectious complications have been reported, as well as a higher rate of post-colectomy reoperation and IPAA failure [150,151,152,153,154,155].

For CD patients, low serum albumin levels seem to correlate with a high risk of post-operative complications with ORs ranging from 1.207 to 2.232, especially septic complications [156,157,158,159,160,161,162,163,164,165]. Even abscess drainage, whether surgical or percutaneous, has a higher risk of complications in such patients (OR 0.921; 95% CI 0.89–0.96) [166].

Post-surgery endoscopic recurrences after initiating anti-TNF therapy are more frequent for albumin values < 3.3 g/dL with an OR of 34.10 (95% CI, 1.72–28.04) [167].

#### 3.4.4. Main Evidence and Clinical Implications

All studies showed that albumin plays a fundamental role in the disease course, response to drugs, surgery rates, and even more concerning the post-surgical complications both in CD and UC patients. Therefore, albumin is a relevant factor to be kept in mind in the management of IBD patients.

## 4. Anemia and Micronutrients

The chronic inflammatory status and the impaired absorption of nutrients due to bowel damage leads to a possible deficiency of vitamins and micronutrients that are crucial for the overall well-being [11]. If the low serum levels of these microelements are a cause or an effect of disease activity remains unclear, and if supplementation of these elements could be a potential therapeutic target is not well defined. However, the evidence is sufficiently solid in showing that active disease is linked to low serum micronutrient levels (Table 3).

The micronutrients and vitamins most involved in IBD are iron, selenium, zinc, copper, manganese, vitamin D, vitamin B12 and folic acid, vitamins A, E, C, K, B1 and B6. Micronutrient and vitamin deficiencies may be linked only to IBD or also to the concomitant presence of other autoimmune diseases such as autoimmune chronic atrophic gastritis (leading to malabsorption of iron and vitamin B12) and celiac disease (responsible for malabsorption of iron and folic acid) [192].

### 4.1. Anemia

Anemia is a common complication of IBD. According to the World Health Organization (WHO) criteria, anemia is defined as a hemoglobin (HgB) level less than 13 g/dL in men and 12 g/dL in non-pregnant females [193].

The most common causes of anemia in IBD are iron deficiency, folic acid or vitamin B12 deficiency, chronic disease anemia due to inflammation, and combined causes. Certainly, it is crucial to classify the etiology of anemia in IBD to select the correct treatment [194,195].

Pure iron deficiency anemia is defined in the case of ferritin serum levels < 30 μg/L and the normal value of CRP; chronic disease anemia is defined by ferritin serum level > 100 μg/L and high levels of CRP. Combined anemia is defined by ferritin serum level < 100 μg/L and high levels of CRP [196].

Anemia affects the quality of life, cognitive functions, the ability to work, hospitalization, and healthcare costs [197]; if low levels of HgB could affect the disease course of IBD is not well known.

#### 4.1.1. Clinical Disease Course and Response to Therapies

A retrospective study on CD patients showed that anemia is a predictive factor for loss of response to anti-TNF [198]; moreover, anemic CD patients had two-times greater odds ratio of severe hospitalization compared with non-anemic patients (OR 1.49, 95% CI: 1.37–1.61) [168].

In UC patients, anemia is a marker of disease severity (e.g., Truelove and Witts index) [199,200], a predictor of relapse in patients treated with 5-ASA, and a predictor of re-admission to hospital, as shown in two large nationwide studies [169,170]. Furthermore, anemia seems to be a predictor of colectomy [171].

Two prospective studies demonstrated that anemia is associated with the extension of the disease, treatment escalation, and disease outcomes (including hospitalization and surgery) in both CD and UC, therefore, its evaluation can be helpful in stratifying disease severity [201,202].

#### 4.1.2. Post-Operative Recurrence and Surgery-Related Complications

Seven retrospective studies analyzed incidence and risk factors for post-operative complications in CD patients undergoing bowel resection, showing that pre-operative anemia was associated with a higher risk of post-operative morbidity and mortality [172,173,174], higher risk of sepsis [160], surgical site infection [175] and post-surgical complications [176] such as prolonged postoperative ileus [177].

#### 4.1.3. Main Evidence and Clinical Implications

- Current evidence suggests that anemia could be associated with high severity of IBD.
- All studies showed a worse disease course or higher risk of post-surgical complications in IBD anemic patients.
- Preoperative correction of anemia may improve surgical outcomes.

### 4.2. Iron

Iron deficiency (ID) is one of the worldwide most common disorders, affecting about 50% of IBD patients [195,203,204], with a prevalence ranging from 26.5% to 62.5% and representing the most common micronutrient deficiency of IBD [195,205,206,207].

In IBD patients, the diagnostic criteria for ID depend on the severity of inflammation: in remission, serum ferritin < 30 µg/L and transferrin saturation index (TSAT) < 16% are indicative of ID, while during the acute phase of the disease (CRP > 5 mg/L and/or FC > 150 mg/kg), ID is defined as ferritin < 100 µg/L.

Iron is an essential trace element involved in many cellular processes including oxygen transport, mitochondrial electron transport, gene regulation and DNA synthesis, and its deficit can manifest through a court of very heterogeneous extra-bowel symptoms including chronic fatigue, sleep disorders, agitation, decreased physical and cognitive performance, immune system impairment, significantly affecting the patient’s wellbeing [178].

Female patients and severe disease activity patients are at higher risk, due to menstrual losses in premenopausal women and to bloody diarrhoea. Interestingly, patients with ID without anaemia presented health-related quality of life (HRQoL) questionnaires with lower overall scores [178,208].

#### 4.2.1. Clinical Disease Course and Response to Therapies

ID in the absence of anaemia negatively impacts the normal perception of HRQoL in patients with IBD, so its early diagnosis and correction could be a valuable target in the treatment of these patients [178,179,180]. Few studies have examined the effects of treating ID in patients with IBD without anaemia using different formulations of intravenous iron and in all of them, there was a significant improvement in patient’ symptoms and HRQoL, independently of disease activity [181,182].

Recently a multicentre, prospective, observational study by Eliadou and colleagues, including 98 IBD patients with ID without anaemia, showed that 1 month after a single 500 mg dose of intravenous ferric carboxymaltose (FCM) there was a significant increase of serum ferritin, serum iron, TSAT, and an improvement of symptoms [209]. Moreover, improvements in the mean EQ-5D scores among CD (*p* < 0.01) and UC (*p* < 0.05) patients were observed [182].

Another single-centre prospective study, including 84 CD patients and 24 healthy volunteers showed that decreased serum iron and total iron binding capacity correlate negatively with CDAI scores (r = −0.513, r = −0.409, both *p* < 0.01), representing independent risk factors for serious disease in a logistic regression analysis, with a sensitivity of 32.7% and a specificity of 100% (AUC = 0.812, *p* < 0.01); a cut-off value of serum iron of 5.25 µmol/L was used to distinguish moderate from mild/remission groups (AUC = 0.729, *p* = 0.001; sensitivity: 48.1%; specificity 93.5%) [181].

There are no definitive recommendations for the treatment of iron deficiency without anaemia (IDWA). Oral intake is not tolerated in up to 20% of patients and could expose patients to adverse effects perceived as symptoms worsening [205,210,211,212,213]. Intravenous iron is more effective, has an excellent security profile and has a reduced risk of adverse events, resulting in better tolerance [214,215].

#### 4.2.2. Main Evidence and Clinical Implications

Iron supply in patients with IBD and IDWA improves the symptoms related to ID and HRQoL.

### 4.3. Vitamin B12 and Folic Acid

Vitamin B12 and folate deficiencies are common in patients with IBD. Folate deficiency is due to a combination of factors: poor diet, malabsorption, an increased requirement due to the increased granulocytes and other inflammatory cells, severe inflammation, resection, enteric fistulas and the use of drugs such as sulfasalazine and methotrexate [216,217].

As shown in a recent meta-analysis, the folate level in IBD patients was significantly lower compared to healthy groups; however, a lower concentration of folate was found in UC but not in CD patients [218].

Vitamin B12 deficiency is more common in CD patients compared to UC, with a prevalence of 33% and 16% respectively [219].

In CD patients, prior intestinal surgery is an independent risk factor for low serum concentrations of vitamin B12 [220]. A meta-analysis by Battat et al. identified that an ileal resection longer than 20 cm is the only factor to predispose CD patients to vitamin B12 deficiency [221].

Vitamin B12 is absorbed in the distal ileum, the intestinal tract most commonly involved in CD, and this would explain the higher prevalence of vitamin B12 deficiency in CD compared to UC.

A periodical assessment of blood levels of vitamins and iron is suggested. Guideline recommendations suggest checking haemoglobin and iron status every 6–12 months for patients in remission or with mild disease, and every 3 months in case of active disease. For patients at risk of vitamin B12 or folic acid deficiency (e.g., small bowel disease or resection), serum levels should be measured at least annually, or when macrocytosis is present [193].

#### Main Evidence and Clinical Implications

Folate deficiency is more common in UC patients while B12 deficiency is more common in CD patients.

### 4.4. Vitamin D

During the last few years, there has been an increase in interest concerning the immuno-modulating role of vitamin D [25,222]. Firstly, several observational and cross-sectional studies included in three meta-analyses showed that IBD patients presented low serum vitamin D levels compared to healthy people [183,223,224]. A meta-regression analysis shows that latitude does not influence the association between IBD and vitamin D deficiency (*p* = 0.34) [223].

#### 4.4.1. Clinical Disease Course and Response to Therapies

An inverse correlation between serum vitamin D levels and disease activity was observed in both UC and CD in all published meta-analyses [183,184]. IBD patients with low vitamin D levels present increased odds of disease activity, mucosal inflammation and future clinical relapse (OR: 1.53, CI 1.32–1.77; OR 1.30, CI 1.06–1.60; OR 1.23, 1.03–1.47, respectively) [184].

However, based on available research it is impossible to understand if vitamin D deficiency is a cause or an effect of disease activity.

Seven RCTs, included in a recent meta-analysis, evaluated disease activity as a dichotomous outcome in IBD patients treated with vitamin D supplementation, showing that the pooled risk ratio of clinical relapse was 0.64 (CI 0.46–0.89) among 458 IBD-treated patients. This data appeared more solid in CD patients in clinical remission (OR 0.47, CI 0.27–0.82). On the other hand, seven studies, included in the same meta-analysis, reported data concerning the impact of vitamin D supplementation on the disease activity score evaluated as a continuous outcome. The authors reported a slight, not statistically significant, effect on CD patients (SMD, −0.29; −0.71–0.14), and no differences in UC (SMD, 0.24, CI −0.61–1.10) [186].

Other four meta-analyses reported data concerning the effectiveness of vitamin D supplementation on disease course in IBD, however, none of these had the clinical relapse or the disease activity as their primary outcome. Guo et al. showed that vitamin D supplementation decreased serum CRP, but it did not decrease the disease activity index and relapse rate. However, only three RCTs were included [225]. Other two meta-analyses including both RCT and observational studies showed an improvement in Harvey Bradshaw index (HBI) of −1.47 points (CI −2.47–−0.47) and a reduction in relapse rate (OR 0.34, CI 0–20–0.58) [226,227].

Another meta-analysis on this topic, including only UC patients, showed a significant reduction in both relapse rate and disease activity, evaluated with the partial Mayo score [228].

In one of the above-mentioned meta-analyses, the authors tried to evaluate the optimal vitamin D dose supplementation with a meta-regression model showing the optimal effect with an intermediate dosage (10,000–15,000 IU/day) [186].

There are four observational studies [24,229,230,231] and two RCTs [232,233] assessing the relationship between vitamin D levels, its supplementation and the response to biologics. Bendix and colleagues showed that a seven weeks high-dose vitamin D treatment reduces the need for later infliximab dose-escalation and reduces inflammatory markers [232]. However, the other RCT did not show a significant reduction in serum TNFα levels [233]. Moreover, all the observational studies showed a significant correlation between low serum vitamin D levels and response to biological therapy. One retrospective study observed a high remission rate, higher reduction in HBI, and increased remission rate, in patients regularly treated with Infliximab and vitamin D supplementation [231]. Additionally, on the multivariate analysis low serum vitamin D levels (<25 ng/mL) were associated with primary non-response to Vedolizumab, failure after one year of follow-up (OR 26.10, CI 14.30–48.90 and OR 6.10, CI 3.06–12.17, respectively) and earlier cessation of anti TNFα therapy (HR 2.13, CI 1.03–4.39) [229,230]. Finally, serum vitamin D levels < 25 ng/mL were associated with loss of response to the biological therapy [24].

#### 4.4.2. Risk of Surgery and Post-Operative Recurrence

Concerning the risk of surgery, a single retrospective study observed that serum vitamin D levels < 20 ng/mL were associated with an increased risk of surgery (OR 1.76, CI 1.24–2.51) and of hospitalization compared to patients with level ≥ 30 ng/mL in both CD and UC [234].

Vitamin D levels > 30 ng/mL were also associated with a reduced risk of endoscopic recurrence in patients who underwent a prior intestinal resection [235], although vitamin D supplementation does not prevent postoperative recurrence in CD patients [185].

#### 4.4.3. Main Evidence and Clinical Implications

- Low serum vitamin D levels are associated with an increase in disease activity and a worse clinical course in IBD patients. Furthermore, vitamin D supplementation results in a slight reduction of clinical relapse. This effect seems to be higher among CD patients.
- Although with low-level evidence, low serum vitamin D levels are associated with an increased risk of surgery.

### 4.5. Other Vitamins (A, E, K, Group B, and C)

As for other nutritional deficits, vitamin deficiency is common in IBD patients, and its pathogenesis is multifactorial [192].

A meta-analysis including 19 case-control studies, showed a lower serum level of fat-soluble vitamins (A, D, E, K) in IBD patients compared to the control group.

Interestingly, in the meta-regression analysis, significant associations between vitamin A levels in CD patients, and the levels of inflammatory biomarkers (CRP: *p* = 0.03, and albumin *p* = 0.0003), were found. The data concerning vitamins E and K were not enough strong to show a correlation with disease activity, however, a clear trend was found [236]. Another study showed a lower level of vitamin K in CD. The vitamin K level (evaluated by measuring serum undercarboxylated osteocalcin) was significantly correlated with the clinical activity index among CD patients [237].

Few studies reported that vitamin C deficiency is relatively common in IBD patients, in particular in patients with reduced intake of vegetables and fruit [238,239].

Little evidence showed a plausible increase in vitamin A levels after adequate treatment for disease activity in UC patients. Plasma vitamin A is significantly lower in active UC patients compared to the control group (*p* = 0.0005) [240,241]. Another retrospective study including CD patients who underwent surgery showed a significantly higher basal peroxidative state and lower levels of Vitamin A and E compared to controls among the CD patients. Two months after surgery, a significant increase in serum vitamin A levels but not Vitamin E was found [242].

Concerning the other vitamin of group B only a few data are available [192,243]. However, some evidence showed that Vitamin B1 deficiency could be related to chronic fatigue in IBD patients [244].

#### 4.5.1. Clinical Disease Course

Vitamin A: The most promising results were observed with vitamin A supplementation. In an RCT including 150 UC pa-tients with moderate-severe disease activity were randomized to receive 25.000 IU/die of Vitamin A or placebo for two months as adjunctive treatment (both groups were treated with 5-ASA for at least a month before study entry). At the end of the follow-up, a significant decrease in the Mayo Clinic score (*p* < 0.001) and sub-scores (*p* < 0.001) was observed in the intervention group. However, another small RCT, published in 1985 showed different results. In this study, there was no correlation between vitamin A levels, and activity indices, and the clinical relapse rate was similar in the two groups [187]. However, a lower relapse rate was observed in the vitamin A-treated group, despite the low sample size included in the study.

Vitamin B: A randomized, double-blinded, placebo-controlled crossover trial including IBD patients with a clinical re-mission evaluated the efficacy of 4 weeks of high-dose oral thiamine (600–1800 mg/dL of B1 vitamin) on chronic fatigue. This study showed a significant reduction in the Inflammatory Bowel Disease-Fatigue Questionnaire during the treatment period [188].

Other promising results derived from a prospective, non-randomized, study of intervention including CD patients treated with 100 mg riboflavin (vitamin B2) daily for 3 weeks. Riboflavin supplementation significantly decreased serum levels of inflammatory markers and the clinical activity evaluated with HBI [189].

#### 4.5.2. Main Evidence and Clinical Implications

- Despite the very low quality of available evidence, Vitamin A and B2 supplementation may have little to no effect in reducing disease activity.
- High dose of Vitamin B1 may reduce chronic fatigue in IBD patients.

### 4.6. Other Trace Elements (Selenium, Zinc, Copper, Manganese)

Zinc and Selenium are involved in the regulation of the immune response, inflammatory processes, and the regulation of oxidative stress [245,246]. Considering this, their low serum concentration may exacerbate inflammation through the dysfunction of the epithelial barrier, an altered mucosal immunity, and an increased production of pro-inflammatory cytokines.

#### 4.6.1. Selenium and Zinc

A study from Siva et al. confirmed that IBD patients with zinc deficiency are more likely to have adverse disease outcomes: a higher risk of surgery, hospitalization, and complications [247].

Evidence on the supplementation of zinc and selenium in IBD is scarce and mostly on mice models. Daily zinc supplementation in CD patients improved intestinal permeability [248], CDAI score and serum zinc level [249]. A randomized, placebo-controlled, trial demonstrated that zinc-carnosine chelate compound enemas used in patients with active UC receiving induction therapy offer a better clinical response or remission than placebo [190]. Concerning selenium, a prospective randomized interventional trial demonstrated that adding Selenium to Infliximab in IBD patients, can reduce adverse drug reactions [250] and can improve clinical symptoms, in patients with mild to moderate UC [191].

#### 4.6.2. Copper and Manganese

Results about serum copper concentrations in IBD patients are not univocal [251,252]. In contrast to zinc, copper concentrations increase during the acute-phase response, resulting in an increased copper/zinc ratio, that should be more relevant than the concentration of copper alone [253].

Although it has an important antioxidant role, manganese is the less studied trace element. An interesting work on IBD patients with ileal pouch-anal anastomosis for a prolonged period showed a significantly increased blood manganese concentration compared to healthy controls. Authors try to interpret this as the result of an increased manganese absorption linked to iron deficiency and the use of antidiarrheal medications [254].

#### 4.6.3. Main Evidence and Clinical Implications

Few data are available concerning the trace elements and their potential impact on the IBD disease course. More systematic screening of trace element status in IBD patients, as well as clinical trials on trace element supplementation are needed.

## 5. Conclusions

**Figure 1:**
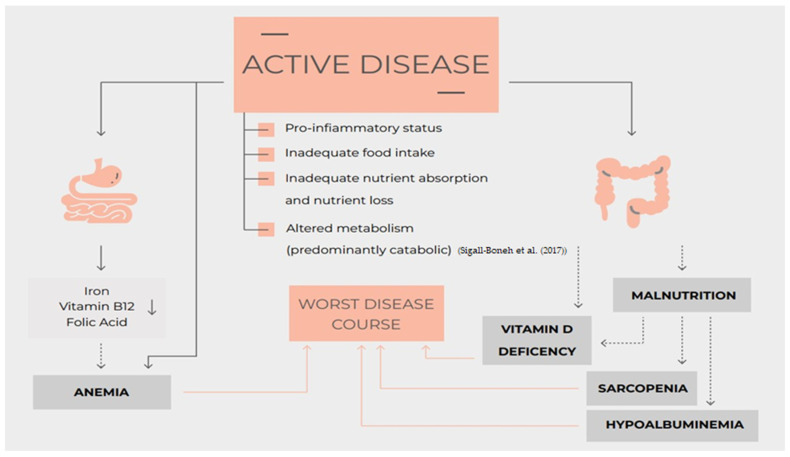
Nutrition, nutritional status, micronutrients deficiency and disease course of Inflammatory Bowel Disease [11].

In a more and more ambitious approach to IBD with a treat-to-target strategy that considered tighter objectives such as mucosal healing, transmural healing, histological healing, and overall, a deep remission of the disease even the nutritional status must be considered [1,2,3,4] (Figure 1).

The evidence summarized in this review showed that many nutritional aspects could be potential targets to induce a better control of symptoms, a deeper remission, and overall improve the quality of life of IBD patients (Table 4).

Certainly, many aspects summarized in this review are still lacking strong evidence. Few data are available concerning the effect of nutritional status on the induction of remission and the impact on the risk of surgery. Obviously, some clinical outcomes need data deriving from RCTs or non-randomized studies of intervention with a long follow-up (such as the risk of surgery, POR, and clinical relapse). Often these data are still lacking. Moreover, considering that the majority of available data derive from observational studies, inclusion criteria, and the analyzed outcome are often heterogeneous.

However, the large number of studies included and analyzed in this review allow us to produce a very extensive overview concerning this issue stating practical clinical aspects and highlighting the current knowledge gap helping to drive future research. An optimal nutritional status and the good management of micronutrient deficiency, ideally with the help of dietitians, may reduce this risk of clinical relapse, risk of surgery and post-operative recurrence. Considering this, all these variables should be considered for the general assessment and monitoring of IBD patients.

## Figures and Tables

**Table 1:** Nutritional support for induction and maintenance of remission in UC and CD.

Nutritional Support	Induction of Remission		Maintenance of Remission	
	UC	CD	UC	CD
EN [32,33,34,35,36,37,38,39,40]	N.A.	Less effective than steroids	N.A.	Higher clinical remission maintenance and lower rate of clinical relapse
Fibres [41,42,43,44]	Better outcome in remission and colectomy rate	No advantage in reducing fibres	Protective effect against relapse	Protective effect against relapse
UCED [45,46]	Higher remission and mucosal healing rates	N.A.	N.A.	N.A.
CDED [47]	N.A.	Effective for induction and maintenance of remission	N.A.	N.A.
Oral diets [27,48]	Not helpful	Higher induction of remission rate	Red meat diet may increase relapse rate; no advantage for other types of diet	No advantage in low red meat and low refined carbohydrates

UC: Ulcerative colitis; CD: Crohn Disease; N.A: Not available; EN: Enteral Nutrition; UCED: Ulcerative Colitis Exclusion Diet; CDED: Crohn’s disease exclusion diet.

**Table 2:** Nutritional support and surgical outcome in UC and CD.

Nutritional Support	Prior to Surgery		Afeter Surgery	
	UC	CD	UC: Pouchitis	CD: Short Bowel Syndrome
EN [52,55]	N.A.	Reduce postoperative complication	Clinical improvement; no improvement in endoscopy and histology	N.A.
Fibres [40,42,43,44,50,51,52,53,54,55,56,57,58]	N.A.	N.A.	Endoscopic and histologic improvement	N.A.
High-carbohydrate-low-fat diet [59]	N.A.	N.A.	N.A.	Carbohydrates in patients without a colon have no effects; fat reduction is not recommended

UC: Ulcerative colitis; CD: Crohn Disease; N.A.: Not available; EN: Enteral Nutrition.

**Table 3:** Anemia, micronutrients deficiency and disease course.

Anemia and Micronutrients Deficiency			
	Effect on Disease Course		Effect of Supplementation
	CD	UC	
Anemia [168,169,170,171,172,173,174,175,176,177]	Higher risk of hospitalization Higher risk of surgery Higher risk of surgery related complication	Higher risk of hospitalization Higher risk of clinical Relapse Higher risk of surgery	N.A.
Iron [178,179,180]	Low QoL Worst disease control	Low QoL	Improve QoL [181,182]
Vitamin B12 and Folic acid	N.A.	N.A.	N.A.
Vitamin D [183,184,185]	Higher risk of active disease Higher risk of clinical relapse Higher risk of surgery	Higher risk of active disease Higher risk of clinical relapse Higher risk of surgery	Lower risk of clinical relapse [186]
Vitamin A	N.A.		Lower risk of clinical relapse [187]
Vitamin B1	N.A.		Reduction of IBD chronic fatigue [188]
Vitamin B2	N.A.		Reduction of clinical activity [189]
Vitamin E, K, C	N.A.		N.A.
Zinc	N.A.		Higher clinical response [190]
Selenium	N.A.		Improve clinical symptoms [191]
Copper and Manganese	N.A.		N.A.

UC: Ulcerative colitis; CD: Crohn Disease; N.A.: Not available; QoL: Quality of life.

**Table 4:** Summary of main findings of nutrition, nutrition status, and micronutrients deficiency.

Nutrition and Nutritional Status					
	Disease Clinical Course	Induction of Remission	Maintenance of Remission	Risk of Surgery	Surgery-Related Complications; POR
Exclusion Diets	Insufficient data	Insufficient data, but most don’t impact	Insufficient data, but most don’t impact	Insufficient data	Insufficient data
EN/PN	Maybe useful	Inferior to steroids	Maybe useful	Insufficient data	Reduce the risk
Sarcopenia	Slight increase in worse clinical and endoscopic outcomes in CD patients	Insufficient data	Slight increase of recurrence after surgery in CD patients.	Slight increase in IBD patients.	Slight increase in POR in CD patients. Higher rate of complications (both in UC and CD patients).
Obesity	Lacking and contrasting data.	May worsen the effectiveness of therapies	Insufficient data	likely increases the risk for surgery (in CD)	increases postoperative complications; risk factor for endoscopic recurrence after surgery (in CD)
Hypoalbuminemia	Probably result in a worst clinical course	Insufficient data	Probably increase the numbers of flares	Result in a large reduction of surgery free survival	Probably increase post-surgical complications
Micronutrients deficiency					
	Disease clinical course	Induction of remission	Maintenance of remission	Risk of surgery	Surgery-related complications; POR
Anaemia	Probably result in a worst clinical course	Insufficient data	Insufficient data	Insufficient data	Probably result in a higher risk of perioperative complication
Iron deficiency	Probably result in a worst clinical course	Insufficient data	Insufficient data	Insufficient data	Insufficient data
Vitamin B12 and Folic acid deficiency	Insufficient data	Insufficient data	Insufficient data	Insufficient data	Insufficient data
Vitamin D deficiency	Probably get worse clinical course	Insufficient data	Increase of clinical relapse	Insufficient data	Insufficient data
Other vitamins deficiency	Vitamin A, B1 and B2 deficiency Probably get worse disease clinical course	Insufficient data	Insufficient data	Insufficient data	Insufficient data
Other micronutrients deficiency	Zinc and selenium probably get worse clinical course	Insufficient data	Insufficient data	Insufficient data	Insufficient data

CD: Crohn Disease; IBD: Inflammatory Bowel Disease; EN/PN: Enteral Nutrition/Parenteral Nutrition; POR: Post-operative recurrence.

## Data Availability

Data sharing not applicable to this article as no datasets were generated or analyzed.

## Supplementary Materials

The following supporting information can be downloaded at: https://www.mdpi.com/article/10.3390/nu15173824/s1, Table S1: Systematic bibliographic research; Table S2: Articles selection process; Figure S1: PRISMA flow diagram.
