# Supplementary material for: Nutrition, Nutritional Status, Micronutrients Deficiency, and Disease Course of Inflammatory Bowel Disease
Source: Nutrients. 2023 Aug 31;15(17):3824. doi: 10.3390/nu15173824 (PMC10489664; doi:10.3390/nu15173824)

Supplementary Table S1. Systematic bibliographic research

- **PubMed**

	**PubMed**
**Nutrition and exclusion diet**	((("inflammatory bowel diseases"[MeSH Terms] OR "crohn disease"[MeSH Terms] OR "colitis, ulcerative"[MeSH Terms]) AND ("nutrition s"[All Fields] OR "nutritional status"[MeSH Terms] OR ("nutritional"[All Fields] AND "status"[All Fields]) OR "nutritional status"[All Fields] OR "nutrition"[All Fields] OR "nutritional sciences"[MeSH Terms] OR ("nutritional"[All Fields] AND "sciences"[All Fields]) OR "nutritional sciences"[All Fields] OR "nutritional"[All Fields] OR "nutritionals"[All Fields] OR "nutritions"[All Fields] OR "nutritive"[All Fields] OR ("diet"[MeSH Terms] OR "diet"[All Fields]) OR "exclusion diet"[All Fields] OR "enteral nutrition"[All Fields]) AND ("clinical relapse"[All Fields] OR "clinical response"[All Fields] OR ("surgery"[MeSH Subheading] OR "surgery"[All Fields] OR "surgical procedures, operative"[MeSH Terms] OR ("surgical"[All Fields] AND "procedures"[All Fields] AND "operative"[All Fields]) OR "operative surgical procedures"[All Fields] OR "general surgery"[MeSH Terms] OR ("general"[All Fields] AND "surgery"[All Fields]) OR "general surgery"[All Fields] OR "surgery s"[All Fields] OR "surgerys"[All Fields] OR "surgeries"[All Fields]))) NOT ("etiology"[MeSH Subheading] OR "etiology"[All Fields] OR "pathogenesis"[All Fields])) AND ((casereports[Filter] OR clinicaltrial[Filter] OR clinicaltrialprotocol[Filter] OR clinicaltrialphasei[Filter] OR clinicaltrialphaseii[Filter] OR clinicaltrialphaseiii[Filter] OR clinicaltrialphaseiv[Filter] OR controlledclinicaltrial[Filter] OR letter[Filter] OR meta-analysis[Filter] OR multicenterstudy[Filter] OR newspaperarticle[Filter] OR observationalstudy[Filter] OR preprint[Filter] OR randomizedcontrolledtrial[Filter] OR systematicreview[Filter]) AND (humans[Filter]))
**Sarcopenia**	(inflammatory bowel disease [MeSH Terms]) OR (inflammatory bowel diseases[MeSH Terms]) OR (IBD) OR (crohn disease[MeSH Terms]) OR (crohn s disease[MeSH Terms]) OR (crohn's disease[MeSH Terms]) OR (crohns disease[MeSH Terms]) OR (CD) OR (colitis, ulcerative[MeSH Terms]) OR (colitis) OR (proctocolitis[MeSH Terms]) OR (proctocolitides, ulcerative[MeSH Terms]) OR (RCU) AND (sarcopenia) OR (sarcopenia[MeSH Terms]) OR (sarcopenias[MeSH Terms])
**Obesity**	(("inflammatory bowel diseases"[MeSH Terms] OR "crohn disease"[MeSH Terms] OR "colitis, ulcerative"[MeSH Terms]) AND (("obesity, abdominal"[MeSH Terms] OR "obesity, abdominal"[MeSH Terms] OR ("obeses"[All Fields] OR "obesity"[MeSH Terms] OR "obesity"[All Fields] OR "obese"[All Fields] OR "obesities"[All Fields] OR "obesity s"[All Fields]) OR "overweight"[MeSH Terms]) AND "humans"[MeSH Terms]) AND "humans"[MeSH Terms] AND ("clinical relapse"[All Fields] OR "clinical response"[All Fields] OR ("surgery"[MeSH Subheading] OR "surgery"[All Fields] OR "surgical procedures, operative"[MeSH Terms] OR ("surgical"[All Fields] AND "procedures"[All Fields] AND "operative"[All Fields]) OR "operative surgical procedures"[All Fields] OR "general surgery"[MeSH Terms] OR ("general"[All Fields] AND "surgery"[All Fields]) OR "general surgery"[All Fields] OR "surgery s"[All Fields] OR "surgerys"[All Fields] OR "surgeries"[All Fields]))) AND (humans[Filter])
**Albuminemia**	("inflammatory bowel diseases"[MeSH Terms] OR ("inflammatory"[All Fields] AND "bowel"[All Fields] AND "diseases"[All Fields]) OR "inflammatory bowel diseases"[All Fields] OR ("inflammatory"[All Fields] AND "bowel"[All Fields] AND "disease"[All Fields]) OR "inflammatory bowel disease"[All Fields] OR "ibd"[All Fields] OR ("crohn disease"[MeSH Terms] OR ("crohn"[All Fields] AND "disease"[All Fields]) OR "crohn disease"[All Fields] OR "crohn s disease"[All Fields]) OR ("colitis, ulcerative"[MeSH Terms] OR ("colitis"[All Fields] AND "ulcerative"[All Fields]) OR "ulcerative colitis"[All Fields] OR ("ulcerative"[All Fields] AND "colitis"[All Fields]))) AND ("serum albumin"[MeSH Terms] OR ("serum"[All Fields] AND "albumin"[All Fields]) OR "serum albumin"[All Fields] OR ("prealbumin"[MeSH Terms] OR "prealbumin"[All Fields] OR "prealbumins"[All Fields]) OR ("hypoalbuminaemia"[All Fields] OR "hypoalbuminemia"[MeSH Terms] OR "hypoalbuminemia"[All Fields])) AND ("colectomy"[MeSH Terms] OR "colectomy"[All Fields] OR "colectomies"[All Fields] OR (("bowel s"[All Fields] OR "bowell"[All Fields] OR "intestines"[MeSH Terms] OR "intestines"[All Fields] OR "bowel"[All Fields] OR "bowels"[All Fields]) AND ("resect"[All Fields] OR "resectability"[All Fields] OR "resectable"[All Fields] OR "resectates"[All Fields] OR "resected"[All Fields] OR "resecting"[All Fields] OR "resection"[All Fields] OR "resectional"[All Fields] OR "resectioned"[All Fields] OR "resectioning"[All Fields] OR "resections"[All Fields] OR "resective"[All Fields] OR "resects"[All Fields])) OR ("stricturoplasties"[All Fields] OR "stricturoplasty"[All Fields]) OR ("recurrence"[MeSH Terms] OR "recurrence"[All Fields] OR "relapse"[All Fields] OR "relapses"[All Fields] OR "relapsing"[All Fields] OR "relapsed"[All Fields] OR "relapser"[All Fields] OR "relapsers"[All Fields]) OR ("loss"[All Fields] AND ("response"[All Fields] OR "responses"[All Fields] OR "responsive"[All Fields] OR "responsiveness"[All Fields] OR "responsivenesses"[All Fields] OR "responsives"[All Fields] OR "responsivities"[All Fields] OR "responsivity"[All Fields])))
**Anemia**	("ibd"[All Fields] OR ("inflammatory bowel diseases"[MeSH Terms] OR ("inflammatory"[All Fields] AND "bowel"[All Fields] AND "diseases"[All Fields]) OR "inflammatory bowel diseases"[All Fields] OR ("inflammatory"[All Fields] AND "bowel"[All Fields] AND "disease"[All Fields]) OR "inflammatory bowel disease"[All Fields]) OR ("crohn disease"[MeSH Terms] OR ("crohn"[All Fields] AND "disease"[All Fields]) OR "crohn disease"[All Fields] OR "crohn s disease"[All Fields]) OR ("colitis, ulcerative"[MeSH Terms] OR ("colitis"[All Fields] AND "ulcerative"[All Fields]) OR "ulcerative colitis"[All Fields] OR ("ulcerative"[All Fields] AND "colitis"[All Fields]))) AND ("colectomy"[MeSH Terms] OR "colectomy"[All Fields] OR "colectomies"[All Fields] OR (("bowel s"[All Fields] OR "bowell"[All Fields] OR "intestines"[MeSH Terms] OR "intestines"[All Fields] OR "bowel"[All Fields] OR "bowels"[All Fields]) AND ("resect"[All Fields] OR "resectability"[All Fields] OR "resectable"[All Fields] OR "resectates"[All Fields] OR "resected"[All Fields] OR "resecting"[All Fields] OR "resection"[All Fields] OR "resectional"[All Fields] OR "resectioned"[All Fields] OR "resectioning"[All Fields] OR "resections"[All Fields] OR "resective"[All Fields] OR "resects"[All Fields])) OR ("stricturoplasties"[All Fields] OR "stricturoplasty"[All Fields]) OR ("recurrence"[MeSH Terms] OR "recurrence"[All Fields] OR "relapse"[All Fields] OR "relapses"[All Fields] OR "relapsing"[All Fields] OR "relapsed"[All Fields] OR "relapser"[All Fields] OR "relapsers"[All Fields]) OR ("loss"[All Fields] AND ("response"[All Fields] OR "responses"[All Fields] OR "responsive"[All Fields] OR "responsiveness"[All Fields] OR "responsivenesses"[All Fields] OR "responsives"[All Fields] OR "responsivities"[All Fields] OR "responsivity"[All Fields]))) AND ("anaemia"[All Fields] OR "anemia"[MeSH Terms] OR "anemia"[All Fields] OR "anaemias"[All Fields] OR "anemias"[All Fields])
**Iron, Vit B 12 and Folic Acid**	(inflammatory bowel disease [MeSH Terms]) OR (inflammatory bowel diseases[MeSH Terms]) OR (IBD) OR (crohn disease[MeSH Terms]) OR (crohn s disease[MeSH Terms]) OR (crohn's disease[MeSH Terms]) OR (crohns disease[MeSH Terms]) OR (colitis, ulcerative[MeSH Terms]) OR (colitis) OR (proctocolitis[MeSH Terms]) OR (proctocolitides, ulcerative[MeSH Terms]) AND (anemia, iron deficiency[MeSH Terms]) OR (anemias, iron deficiency[MeSH Terms]) OR (sideremia) OR ("blood iron") OR (iron) OR (deficiencies, vitamin b12[MeSH Terms]) OR ("vitamin b12") OR (cobalamin) OR (cobalamin[MeSH Terms]) OR (cobalamins[MeSH Terms]) OR (acid deficiency, folic[MeSH Terms]) OR ("folic acid") OR (folic) OR (folate) OR (folate[MeSH Terms]) OR (vitamin b9[MeSH Terms]) OR ("vitamin b9") AND ("clinical relapse") OR ("clinical response") OR (surgery) OR (relapse)<br>Filters: Humans
**Vitamin D**	("IBD"[All Fields] OR ("crohn"[All Fields] OR "crohn s"[All Fields] OR "crohns"[All Fields]) OR ("colitis, ulcerative"[MeSH Terms] OR ("colitis"[All Fields] AND "ulcerative"[All Fields]) OR "ulcerative colitis"[All Fields] OR ("ulcerative"[All Fields] AND "colitis"[All Fields])) OR ("colitis"[MeSH Terms] OR "colitis"[All Fields] OR "colitides"[All Fields]) OR ("inflammatory bowel diseases"[MeSH Terms] OR ("inflammatory"[All Fields] AND "bowel"[All Fields] AND "diseases"[All Fields]) OR "inflammatory bowel diseases"[All Fields] OR ("inflammatory"[All Fields] AND "bowel"[All Fields] AND "disease"[All Fields]) OR "inflammatory bowel disease"[All Fields]) OR "inflammatory bowel diseases"[MeSH Terms] OR "crohn disease"[MeSH Terms] OR "crohn disease"[MeSH Terms] OR "crohn disease"[MeSH Terms] OR "crohn disease"[MeSH Terms] OR "colitis, ulcerative"[MeSH Terms] OR "proctocolitis"[MeSH Terms] OR "RCU"[All Fields]) **AND** ("vitamin D"[All Fields] OR ("cholecalciferol"[MeSH Terms] OR "cholecalciferol"[All Fields] OR "cholecalciferols"[All Fields] OR "colecalciferol"[All Fields]) OR ("cholecalciferol"[MeSH Terms] OR "cholecalciferol"[All Fields] OR "cholecalciferols"[All Fields] OR "colecalciferol"[All Fields]) OR ("cholecalciferol"[MeSH Terms] OR "cholecalciferol"[All Fields] OR ("vitamin"[All Fields] AND "d3"[All Fields]) OR "vitamin d3"[All Fields]) OR "cholecalciferol"[MeSH Terms])
**Other vitamins**	("inflammatory bowel diseases"[MeSH Terms] OR "inflammatory bowel diseases"[MeSH Terms] OR "IBD"[All Fields] OR "crohn disease"[MeSH Terms] OR "crohn disease"[MeSH Terms] OR "crohn disease"[MeSH Terms] OR "crohn disease"[MeSH Terms] OR "colitis, ulcerative"[MeSH Terms] OR ("colitis"[MeSH Terms] OR "colitis"[All Fields] OR "colitides"[All Fields]) OR "proctocolitis"[MeSH Terms] OR "proctocolitis"[MeSH

	Terms]) AND ("vitamin a"[MeSH Terms] OR "vitamin e deficiency"[MeSH Terms] OR "ascorbic acid deficiency"[MeSH Terms] OR "vitamin k deficiency"[MeSH Terms] OR "thiamine"[MeSH Terms] OR "vitamin b 6 deficiency"[MeSH Terms])
**Other micronutrients**	("inflammatory bowel diseases"[MeSH Terms] OR "inflammatory bowel diseases"[MeSH Terms] OR "IBD"[All Fields] OR "crohn disease"[MeSH Terms] OR "crohn disease"[MeSH Terms] OR "crohn disease"[MeSH Terms] OR "crohn disease"[MeSH Terms] OR "colitis, ulcerative"[MeSH Terms] OR ("colitis"[MeSH Terms] OR "colitis"[All Fields] OR "colitides"[All Fields]) OR "proctocolitis"[MeSH Terms] OR "proctocolitis"[MeSH Terms]) AND ("zinc compounds"[MeSH Terms] OR "manganese compounds"[MeSH Terms] OR "copper"[MeSH Terms] OR "selenium"[MeSH Terms])

- **Scopus**

	**Scopus**
**Nutrition and exclusion diet**	TITLE-ABS-KEY (("infammatory bowel disease" OR "Ulcerative colitis" OR "crohn disease" OR ibd) AND (nutrition OR "enteral nutrition" OR "exclusion diet") AND ("clinical response" OR "clinical relapse" OR surgery)) AND (LIMIT-TO (DOCTYPE , "ar")) AND (LIMIT-TO (EXACTKEYWORD , "Human")) AND (LIMIT-TO (LANGUAGE , "English"))
**Sarcopenia**	(TITLE-ABS-KEY ("inflammatory bowel disease") OR ("ulcerative colitis") OR ("crohn disease") OR (rcu) (ibd) OR (cd) AND (sarcopenia))
**Obesity**	TITLE-ABS-KEY (("infammatory bowel disease" OR "Ulcerative colitis" OR "crohn disease" OR ibd) AND (obesity OR central AND obesity OR abdominal AND obesity OR overweight) AND ("clinical response" OR "clinical relapse" OR surgery))
**Albuminemia**	(((inflammatory bowel disease) OR (ibd) OR (crohn's disease) OR (ulcerative colitis)) AND ((serum albumin) OR (prealbumin) OR (hypoalbuminemia)) AND ((colectomy) OR (bowel resection) OR (stricturoplasty) OR (relapse) OR (loss of response)))
**Anemia**	((ibd) OR (inflammatory bowel disease) OR (crohn's disease) OR (ulcerative colitis)) AND ((colectomy) OR (bowel resection) OR (stricturoplasty) OR (relapse) OR (loss of response)) AND (deficiency anemia)
**Iron, Vit B 12 and Folic Acid**	TITLE-ABS-KEY ("inflammatory bowel disease") OR ("ulcerative colitis") OR (crohn) OR ("crohn disease")) AND ((iron) OR ("vitamin b12") OR cob(alamin) OR ("folic acid") OR (folate) OR (folic) OR ("vitamina b9")) AND (("clinical relapse") OR (surgery) OR ("clinical response"))
**Vitamin D**	(((inflammatory bowel disease) OR (ibd) OR (crohn's disease) OR (ulcerative colitis)) AND ((Vitamin D) OR (cholecalciferol) OR (25OHD) OR (cholecalciferol)) AND ((colectomy) OR (bowel resection) OR (stricturoplasty) OR (relapse) OR (loss of response)))
**Other vitamins**	("inflammatory bowel disease" OR ibd OR "crohn's disease" OR "ulcerative colitis" AND "Vitamin A" OR "Vitamin B1" OR "Vitamin B6" OR "vitamin E" OR "vitamin K" OR "vitamin C" AND "bowel resection" OR "stricturoplasty" OR "clinical relapse" OR "loss of response" OR "post operative recurrence" OR "surgery")
**Other micronutrients**	("inflammatory bowel disease" OR ibd OR "crohn's disease" OR "ulcerative colitis" AND "Zinc" OR "selenium" OR "copper" OR "manganese" AND "bowel resection" OR "stricturoplasty" OR "clinical relapse" OR "loss of response" OR "post operative recurrence" OR "surgery")

Supplementary Table S2. Articles selection process

	**Records identified (n PubMed + n Scopus)**	**Records after duplicates removed**	**Articles assessed for eligibility**	**Records identified from other sources**	**Full-text articles included**
**Nutrition and exclusion diet**	895 (334+561)	854	60	0	32
**Sarcopenia**	355 (156+199)	243	55	1	38
**Obesity**	659 (258+401)	522	65	0	18
**Albuminemia**	687 (273+414)	489	64	0	56
**Anemia**	555 (333+222)	494	32	0	15
**Iron, Vit B 12 and Folic Acid**	836 (434+402)	753	51	0	6(B12 and folic acid) +18 (Iron)
**Vitamin D**	1500 (1224+276)	1385	44	0	18
**Other vitamins**	305 (245+60)	298	18	1	14
**Other micronutrients**	285 (185+100)	277	23	0	12
**Total**	6077 (3442+2634)	5315	412	2	227

Supplementary Figure S1. PRISMA Flow diagram

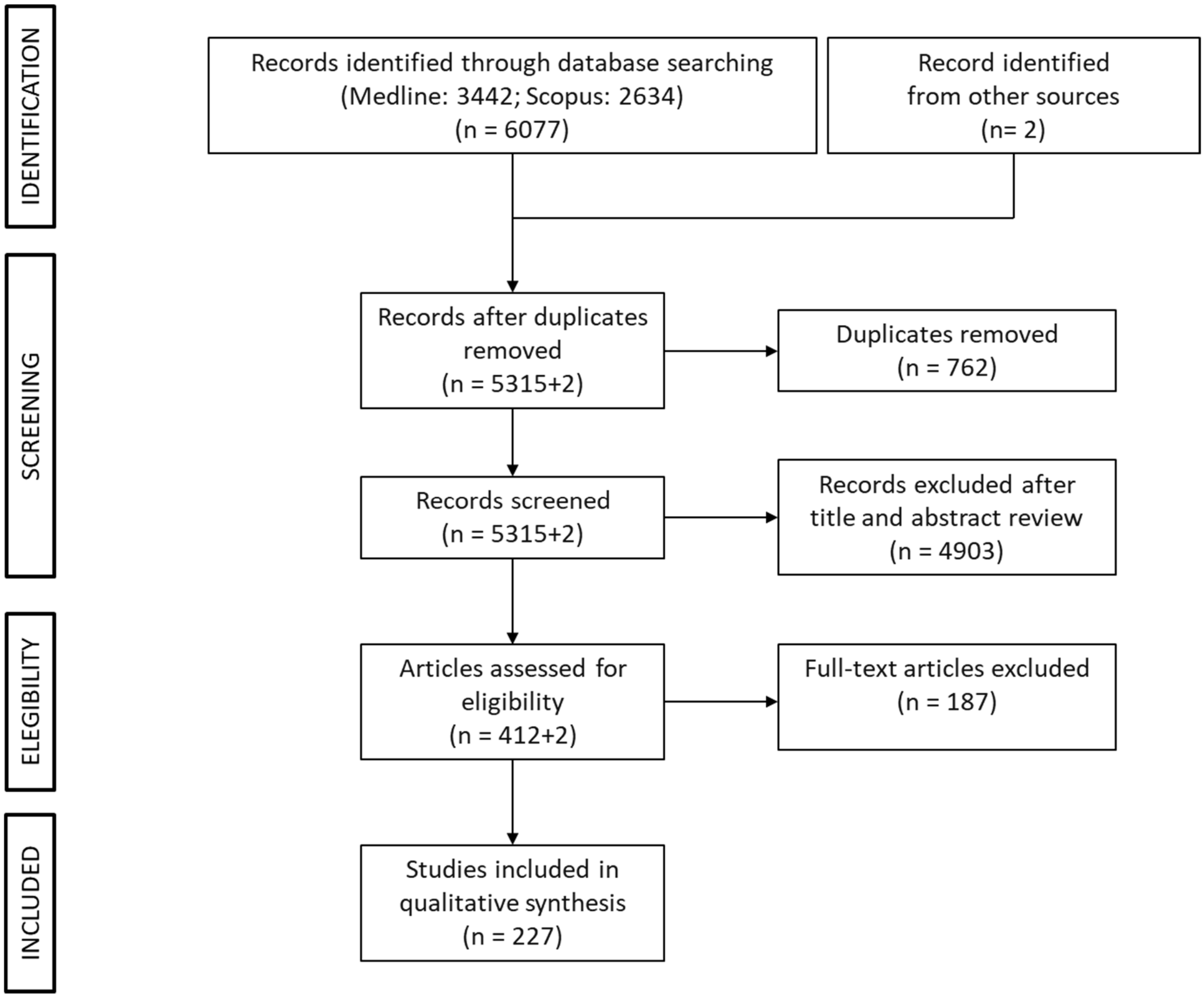

Supplement: Supplementary file 1 [file nutrients-15-03824-s001.zip › nutrients-2579525-supplementary.pdf]
